# Molecular Tuning of IR-786 for Improved Brown Adipose Tissue Imaging

**DOI:** 10.3390/ijms232213756

**Published:** 2022-11-09

**Authors:** Gayoung Jo, Eun Jeong Kim, Juhyun Song, Hoon Hyun

**Affiliations:** 1 Department of Biomedical Sciences, Chonnam National University Medical School, Hwasun 58128, Korea; 2 Department of Anatomy, Chonnam National University Medical School, Hwasun 58128, Korea; 3 BioMedical Sciences Graduate Program (BMSGP), Chonnam National University, Hwasun 58128, Korea

**Keywords:** brown adipose tissue, near-infrared fluorescence imaging, heptamethine cyanine dyes, targeted imaging, IR-786

## Abstract

To overcome the limitations of brown adipose tissue (BAT) imaging with MRI and PET/CT, near-infrared (NIR) fluorescence imaging has been utilized in living animals because it is highly sensitive, noninvasive, nonradioactive, and cost-effective. To date, only a few NIR fluorescent dyes for detecting BAT have been reported based on the structure-inherent targeting strategy. Among them, IR-786, a commercial cyanine dye, was used firstly for quantitative NIR imaging of BAT perfusion in 2003. Owing to the high cytotoxicity, poor water solubility, and strong nonspecific background uptake of IR-786, the chemical structure of IR-786 should be redesigned to be more hydrophilic and less toxic so that it can show more BAT-specific accumulation. Here, we developed a BAT-specific NIR dye, BF800-AM, by incorporating the tyramine linker in the original structure of IR-786. After modifying the physicochemical properties of IR-786, in vivo results showed significant uptake of the newly designed BF800-AM in the BAT with improved signal-to-background ratio. Additional in vivo studies using mouse tumor models revealed that BF800-AM targeting to BAT is independent of tumor tissues, as distinct from IR-786 showing uptake in both tissues. Therefore, BF800-AM can be used for improved noninvasive visualization of BAT mass and activity in living animals.

## 1. Introduction

Brown adipose tissue (BAT) plays an important role in metabolism and thermoregulation, because BAT contains a large number of mitochondria to dissipate chemical energy, abundant uncoupling protein-1 to mediate adaptive nonshivering thermogenesis and metabolic inefficiency, as well as highly vascularized vessel structures to promote efficient heat transfer to the bloodstream [1,2,3]. Owing to the increasing recognition of the role of BAT in diverse physiological processes, noninvasive imaging modalities such as magnetic resonance imaging (MRI), positron emission tomography (PET), and computed tomography (CT) have typically been used to assess the presence and activity of BAT. Previously, PET/CT imaging studies have reported that BAT mass is inversely correlated with body mass index and other obesity parameters in human adults [4,5]. However, BAT is still present in adults in the upper chest, neck, and in other locations. In this regard, BAT has emerged as an important target for the treatment of obesity and diabetes by exploring strategies to activate and increase BAT mass, including “browning” of white adipose tissue (WAT), BAT transplantation, and BAT mass promotion by drug treatment [5].

Although the most common in vivo imaging modalities for BAT imaging are clinically available MRI and PET/CT, these imaging techniques have several drawbacks, including low resolution and sensitivity, the high cost of equipment, and the use of radioactivity; in particular, BAT imaging with ^18^F-FDG for a PET scan requires pretreatment activation such as cold stress or norepinephrine treatment [5]. To overcome those limitations, near-infrared (NIR) fluorescence imaging has been widely utilized in living animals because it is highly sensitive, noninvasive, nonradioactive, and cost-effective [6]. Particularly, the NIR fluorescence imaging system is more suitable for preclinical in vivo screening in small animals. For the NIR fluorescence BAT imaging, mouse interscapular BAT is ideally located away from major organs such as heart, lungs, liver, and stomach to avoid signal overlap, and also has a distinct triangular contour which is easy to distinguish from other tissues [6].

To date, several NIR fluorescence imaging agents have been developed for detecting BAT in small animals. Azhdarinia et al. reported a peptide-based BAT-targeting agent that could be used for BAT imaging after fluorescent labeling with a commercial NIR dye IRDye800CW [7]. Rice et al. also reported a lipophilic and uncharged squaraine rotaxane probe, SRFluor680, which is a commercially available NIR fluorescent probe that could be employed for selective interscapular BAT-targeting in mice using a micellar formulation of SRFluor680 [4]. Additionally, Guo et al. developed NIR fluorescent polymer dots for whole-body BAT imaging in rodents [8]. In terms of the structure-inherent targeting strategy using a small-molecule dye, only three NIR fluorescent dyes for BAT imaging have been reported: (1) a curcumin analogue CRANAD-29 was developed by Zhang et al. through a top-down imaging screening of 38 fluorescent dyes that could be used for imaging BAT and monitoring “browning” of WAT [5], (2) a heptamethine cyanine dye CyHF-8 was synthesized by Li et al. through the quaternization reaction of 2,3,3-trimethylindolenine with tetrahydrofurfuryl bromide to be accumulated in the subcellular mitochondria of brown adipocytes and to noninvasively assess BAT metabolism in living animals [9], and (3) a commercially available heptamethine cyanine dye IR-786 was utilized by Nakayama et al. for the first time to measure BAT perfusion in transgenic models of adaptive thermogenesis in 2003 [6]. Still, reliable and easily synthesized NIR dyes to use for improved BAT imaging are highly desired.

**Scheme 1:**
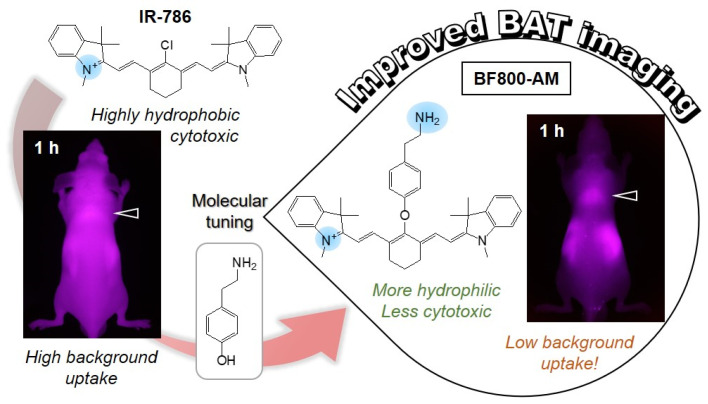
Schematic illustration of the molecular tuning of IR-786 for improved BAT imaging. The introduction of a tyramine linker on the heptamethine core is the key to improving noninvasive NIR fluorescence imaging of BAT owing to the low nonspecific background uptake.

Among the BAT imaging dyes, a lipophilic cationic heptamethine cyanine dye IR-786, known as a mitochondria-targeted NIR dye, was previously redesigned by our group to be more accumulated in the tumor tissue through functionalization of IR-786 with two carboxyl groups [10]. Thus, the chemical structure of IR-786 should be modified to be more hydrophilic and less cytotoxic so that it can be used for improved BAT imaging. In this study, we developed a BAT-specific NIR dye, named BF800-AM, by incorporating the tyramine linker in the original structure of IR-786. To improve the physicochemical properties and in vivo performance of IR-786, the tyramine linker substitution on the chloro-cyclohexenyl ring of IR-786 is a simple and effective approach to increase the BAT selectivity with low nonspecific background uptake (Scheme 1). To the best of our knowledge, no study has investigated the effect of chemically modified IR-786 on the BAT-selective imaging in normal mice and tumor-bearing mice, respectively. Therefore, this study provides a facile molecular tuning strategy to enhance the target-specificity, water solubility, and biocompatibility for future clinical use.

## 2. Results

### 2.1. Synthesis and Characterization of BF800 NIR Dyes

**Figure 1:**
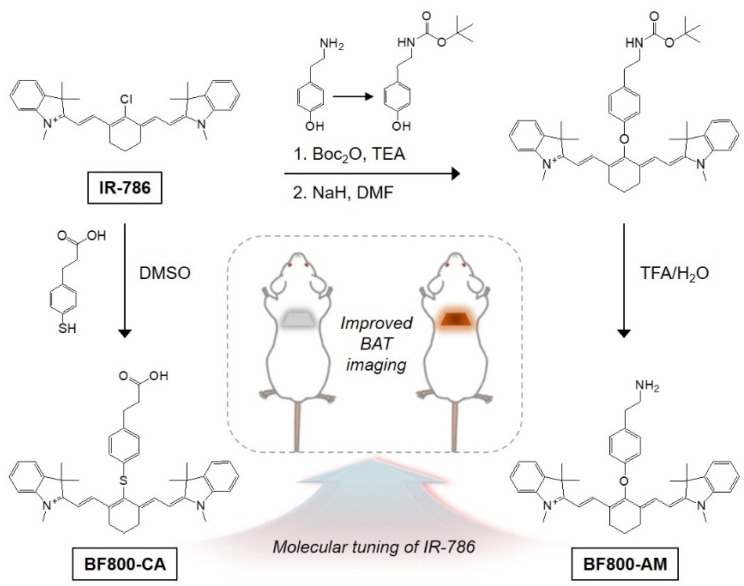
Synthetic scheme of BF800-AM and BF800-CA NIR dyes by the molecular tuning strategy for improved BAT imaging.

BF800-AM designed from the commercial NIR dye IR-786 was synthesized by the isosteric substitution of a meso-chlorine atom on the IR-786 skeleton with a tyramine linker (Figure 1). To prevent an unwanted reaction between the amine group of tyramine and the chloro-cyclohexenyl ring of IR-786, the tert-butoxycarbonyl (Boc) group was used to protect the amine group in the tyramine structure before proceeding further. Subsequently, the substitution reaction occurred by the nucleophilic attack of the phenoxide ion of Boc-protected tyramine to the chloro-cyclohexene on the heptamethine chain of IR-786. Based on the reaction mechanism, sodium hydride was employed to produce the phenoxide ions to increase the nucleophilicity of Boc-protected tyramine. Finally, BF800-AM was readily obtained by removal of the Boc-protecting group after treatment with a solution of trifluoroacetic acid (TFA) and water. Another IR-786 derivative, BF800-CA, containing the carboxylate linker was synthesized by application of the S_RN_1 reaction. To introduce a sulfide linkage on the heptamethine bridge of IR-786, 3-(4-mercaptophenyl)propionic acid was used directly to produce zwitterionic BF800-CA. The chemical structure of BF800-CA containing a sulfide linkage, instead of an ether linkage, was designed to avoid the similar structure of CTNF103, which was previously developed by Park et al. [11].

**Figure 2:**
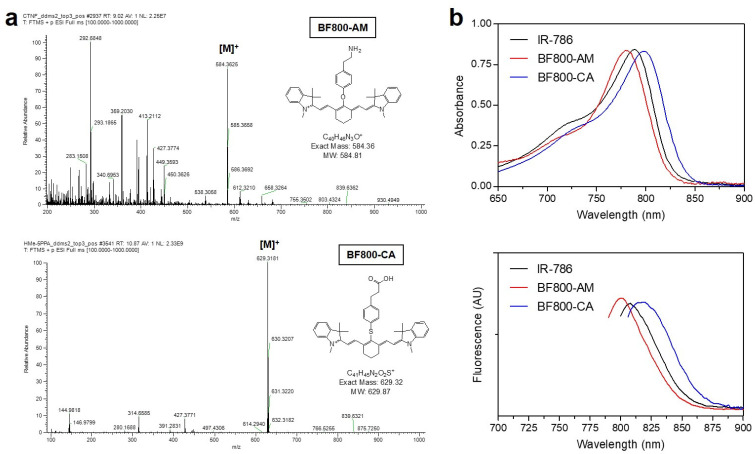
(**a**) Mass spectra of BF800-AM and BF800-CA NIR dyes. (**b**) Absorption spectra and fluorescence emission spectra of IR-786, BF800-AM, and BF800-CA NIR dyes. Optical measurements were performed in PBS at pH 7.4 or 5% BSA saline.

For further use of BF800 NIR dyes in in vitro and in vivo studies, the exact molecular weights of BF800-AM and BF800-CA were successfully identified by mass spectrometry (Figure 2a). The absorption and fluorescence emission peaks of IR-786, BF800-AM, and BF800-CA were consistently displayed in the NIR spectral region (780–820 nm) and exhibited 18–20 nm Stokes shifts, respectively, compatible with the NIR fluorescence imaging system (Figure 2b). As previously reported, the oxygen (BF800-AM) and sulfur (BF800-CA) atoms of the BF800 dyes, after the isosteric replacement of the meso-chlorine atom of IR-786, participate in the intermolecular charge transfers that affords the absorption red/blue shifts [12].

**Figure 3:**
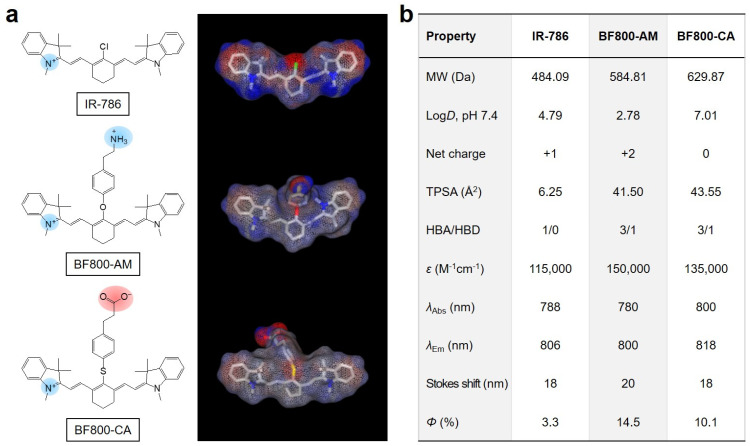
(**a**) Chemical structure, net surface charge, and three-dimensional (3D) modeling of the geometrical positions of IR-786, BF800-AM, and BF800-CA NIR dyes. Red, negative charge; blue, positive charge; gray, hydrophobicity. (**b**) Physicochemical and optical properties of IR-786, BF800-AM, and BF800-CA NIR dyes. In silico calculations of the distribution coefficient (log*D* at pH 7.4), topological polar surface area (TPSA), and hydrogen bond acceptors/donors (HBA/HBD) were performed using Marvin and JChem calculator plugins (ChemAxon).

The chemical structures and 3D energy-minimized structures of all three NIR dyes are shown in Figure 3a to compare the different distributions of charge and hydrophobicity over the surface of each dye. Moreover, in silico predictions of the physicochemical properties including hydrophobicity (log*D* at pH 7.4), polarity (TPSA), surface charge, and hydrogen bonding interactions (HBA/HBD), as well as the optical properties such as molar extinction coefficient and quantum yield of IR-786 and BF800 dyes were performed using JChem (ChemAxon) (Figure 3b). Taken together, BF800-AM displayed better physicochemical properties, such as hydrophilicity (log*D* = 2.78) and polarity (TPSA = 41.50 Å^2^), and optical properties, such as molar extinction coefficient (*ε*_(780nm)_ = 150,000 M^−1^cm^−1^) and quantum yield (Φ = 14.5%) compared to that of IR-786 (4.79, 6.25 Å^2^, 115,000 M^−1^cm^−1^, and 3.3%, respectively). Although the zwitterionic BF800-CA also exhibited improved properties in terms of TPSA (43.55 Å^2^), molar extinction coefficient (*ε*_(800nm)_ = 135,000 M^−1^cm^−1^) and quantum yield (Φ = 10.1%) compared to that of IR-786, the increased hydrophobicity (log*D* = 7.01) of BF800-CA may contribute to high cytotoxicity and low BAT uptake.

### 2.2. In Vitro Cytotoxicity and Cellular Uptake

**Figure 4:**
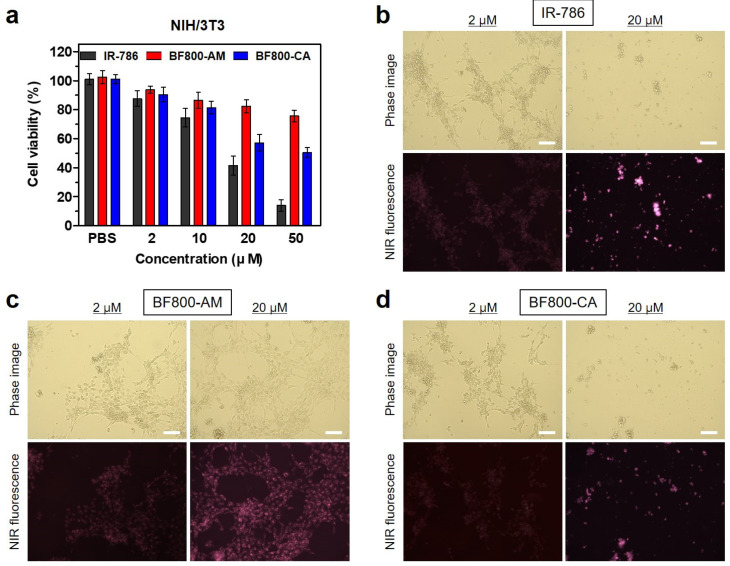
(**a**) Cell viability assay of each NIR dye using NIH/3T3 cells. Percentage cytotoxicity was determined after 24 h of treatment with various concentrations of each NIR dye. Live cell binding of (**b**) IR-786, (**c**) BF800-AM, and (**d**) BF800-CA NIR dyes in NIH/3T3 cells. The phase contrast and NIR fluorescence images of the cell line were obtained using 2 and 20 µM of each NIR dye. Images are representative of three independent experiments. All NIR fluorescence images have identical exposure times and normalization. Scale bars = 100 μm.

The in vitro cytotoxicity was evaluated using the MTT assay after incubation of the NIH/3T3 cells with various concentrations of IR-786, BF800-AM, and BF800-CA for 24 h (Figure 4a). As expected, the cell viability of IR-786 decreased severely in higher concentrations than 10 µM, which is consistent with the previous study. Importantly, BF800-AM showed high average cell viability levels in the concentration range from 2–50 μM, possibly owing to its improved water solubility. However, some cytotoxicity for BF800-CA was observed with an increase in concentration. This result indicates that BF800-AM has good biocompatibility, which is suitable for in vivo applications, overcoming one of the major drawbacks of IR-786. Additionally, we observed the intracellular uptake of each dye after 24 h of incubation in NIH/3T3 cells (Figure 4b–d). Fluorescence signals of each dye were detected in the cells after 24 h of treatment and showed in a concentration-dependent manner. Although all three dyes displayed intracellular uptake with significant fluorescence signals in the cytoplasm, reduced cell proliferation was observed in the 20 μM concentrations of IR-786 and BF800-CA, which corresponded to the results of cell viability tests. Interestingly, BF800-AM exhibited stable cell growth under the same condition, due to the influence of tyramine linker substitution on the IR-786 skeleton, which could improve the water solubility and cytocompatibility.

### 2.3. Time-Dependent In Vivo BAT Imaging and Biodistribution

**Figure 5:**
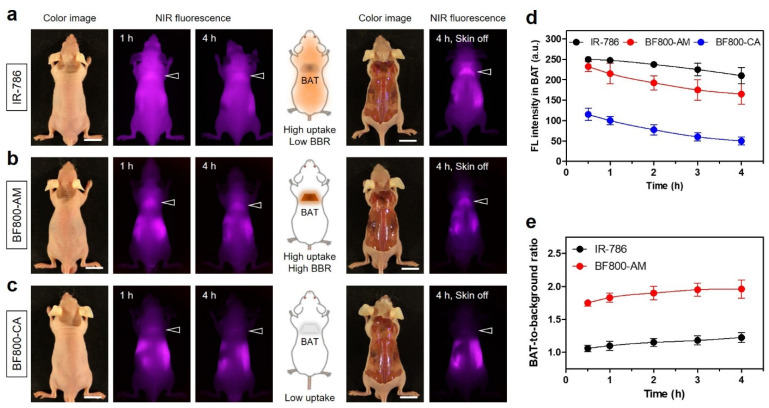
In vivo BAT-targeting efficiency of IR-786, BF800-AM, and BF800-CA NIR dyes. NIR fluorescence imaging 1 and 4 h after injection of (**a**) IR-786, (**b**) BF800-AM, and (**c**) BF800-CA NIR dyes. (**d**) Time-dependent fluorescence intensities at the BAT sites targeted by IR-786, BF800-AM, and BF800-CA NIR dyes. (**e**) Time course of BAT-to-background ratio (BBR) for 4 h post-injection of IR-786 and BF800-AM. BBR was calculated as the fluorescence intensity of BAT versus the fluorescence intensity of neighboring tissue obtained at different time points. The BAT sites are indicated by arrowheads. Scale bars = 1 cm. Images are representative of three independent experiments. All NIR fluorescence images have identical exposure times and normalization.

After confirming the good biocompatibility of BF800-AM in vitro, the BAT-specific targeting of BF800-AM in vivo was further investigated in nude mice to monitor the interscapular BAT in real-time. IR-786, BF800-AM, and BF800-CA were intravenously injected into nude mice and real-time NIR fluorescence imaging was subsequently performed for 4 h post-injection. Although the strong fluorescence signal from the interscapular BAT injected with IR-786 was observed until 4 h post-injection, the high nonspecific background fluorescence signal surrounding the BAT was also maintained (Figure 5a,d). Hence, the shape and mass of BAT can be clearly identified only after skin removal, because of the disturbing background signals. The fluorescence intensity at the BAT site treated with BF800-AM gradually decreased for 4 h after administration (Figure 5b,d). However, BF800-AM showed better BAT-specific imaging with reduced nonspecific background fluorescence compared to that of IR-786. Thus, the shape and mass of BAT can be distinguished without skin removal owing to the low background uptake of BF800-AM. Interestingly, BF800-CA exhibited poor BAT uptake even at 1 h post-injection and the fluorescence signal in BAT was almost undetectable even after skin removal at 4 h post-injection (Figure 5c,d). This indicates that the carboxyl group of the BF800-CA structure contributed to the surface charge compensation, thereby resulting in low BAT uptake. In terms of the BAT-to-background ratio (BBR), BF800-AM is more useful for noninvasive BAT-specific imaging because of its higher BBR than that of IR-786 (Figure 5e).

**Figure 6:**
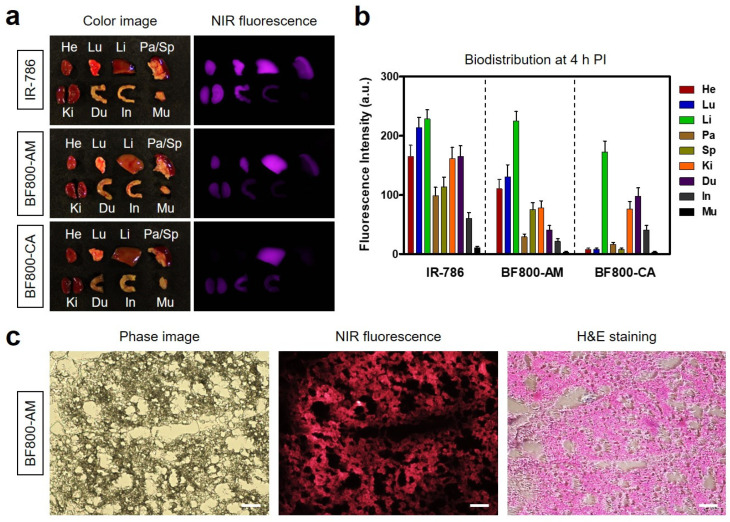
In vivo biodistribution of IR-786, BF800-AM, and BF800-CA NIR dyes. (**a**) Resected organs imaged 4 h after injection of each NIR dye. (**b**) Quantitative fluorescence analysis of intraoperative dissected organs 4 h post-injection of each NIR dye. Abbreviations: Du, duodenum; He, heart; In, intestines; Ki, kidneys; Li, liver; Lu, lungs; Mu, muscle; Pa, pancreas; Sp, spleen; PI, post-injection. (**c**) Phase contrast imaging, NIR fluorescence imaging, and H&E staining of the resected BAT sample 4 h after injection of BF800-AM. Scale bars = 100 μm. Images are representative of three independent experiments. All NIR fluorescence images have identical exposure times and normalization.

To investigate the biodistribution and clearance of each dye, the major organs including heart, lung, liver, pancreas, spleen, and kidney were collected 4 h after injection to confirm their fluorescence signals (Figure 6a). As expected, IR-786 showed high nonspecific uptake in all major organs in the body at 4 h after injection. Although BF800-AM also exhibited nonspecific uptake in the major organs except for the pancreas, the fluorescence intensities in the heart, lung, spleen, and kidney were diminished except for liver tissue, compared to that of IR-786 (Figure 6b). However, BF800-CA displayed no uptake in the heart, lung, pancreas, and spleen at 4 h after injection only with weak fluorescence signals in the liver and kidney. This suggests that the surface charge compensation of the BF800-CA structure may help to reduce the nonspecific organ/tissue uptake. The BF800-AM and BF800-CA derived from the IR-786 structure showed better distribution and clearance from the major organs, thereby resulting in reduced nonspecific background uptake. Additionally, histological sections were obtained from the BAT samples targeted by BF800-AM, observed by fluorescence microscopy, and subsequently stained with H&E (Figure 6c). The phase and H&E images revealed the characteristic features of small lipid droplets with extensive vasculature for BAT. Importantly, the histological analysis using NIR fluorescence microscopy demonstrated that BF800-AM was specifically accumulated in the BAT with strong NIR fluorescence.

### 2.4. In Vivo BAT-Targeting Specificity

**Figure 7:**
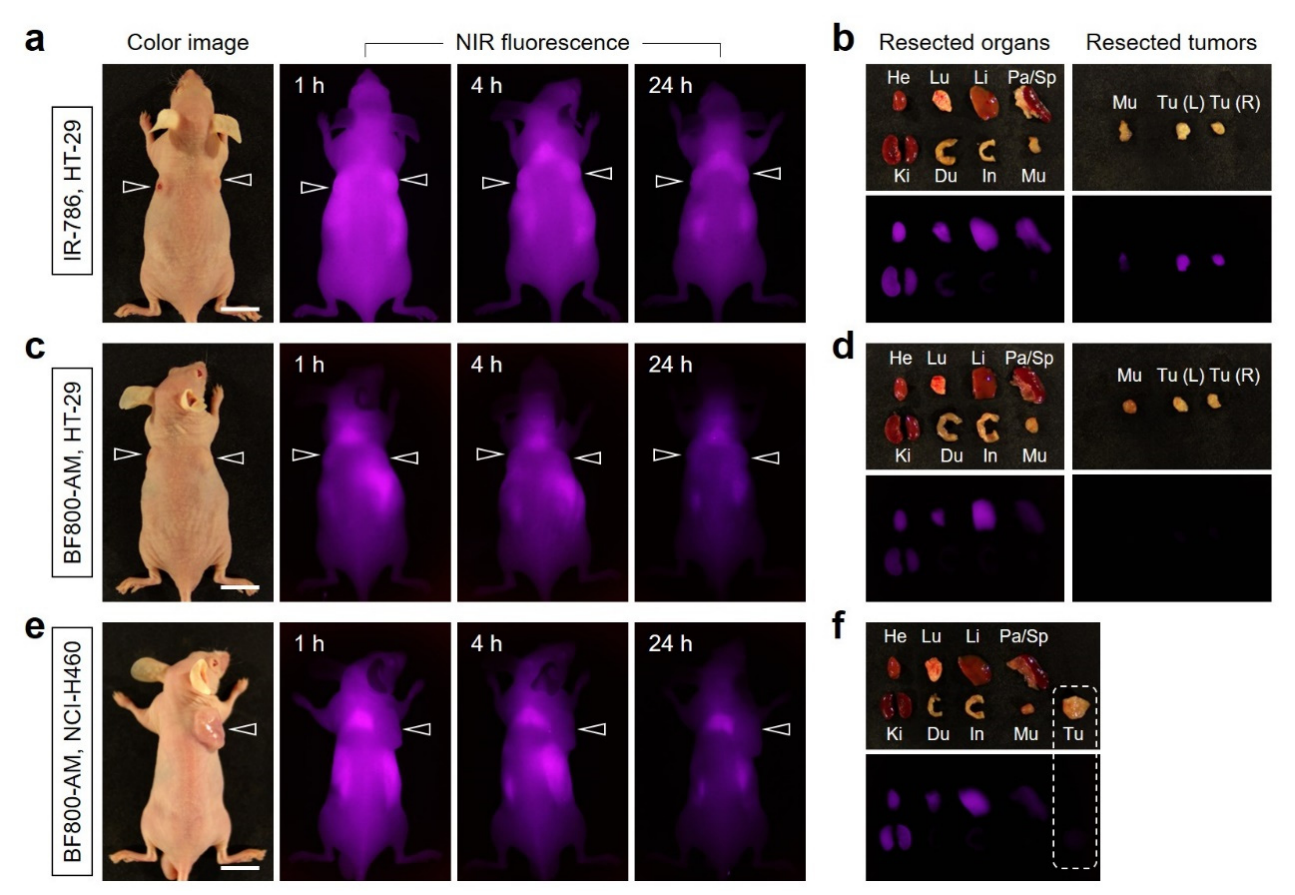
In vivo BAT and/or tumor uptake of IR-786 and BF800-AM in HT-29 or NCI-H460 tumor-bearing mice. Time-dependent NIR fluorescence imaging 24 h after injection of (**a**) IR-786 and (**c**) BF800-AM in HT-29 tumor-bearing mice. Resected organs and tumors imaged 24 h after injection of (**b**) IR-786 and (**d**) BF800-AM. (**e**) Time-dependent NIR fluorescence imaging 24 h after injection of BF800-AM in NCI-H460 tumor-bearing mice. (**f**) Resected organs and tumors imaged 24 h after injection of BF800-AM. The tumor sites are indicated by arrowheads. Abbreviations: Du, duodenum; He, heart; In, intestines; Ki, kidneys; L, the left; Li, liver; Lu, lungs; Mu, muscle; Pa, pancreas; R, the right; Sp, spleen; Tu, tumor. Scale bars = 1 cm. Images are representative of three independent experiments. All NIR fluorescence images have identical exposure times and normalization.

To evaluate the in vivo BAT-targeting specificity in a xenograft mouse model, IR-786 and BF800-AM were intravenously administrated to HT-29 tumor-bearing mice. Since IR-786 is known to show nonspecific tumor uptake, it is highly important to identify whether BF800-AM could be available only for in vivo BAT-specific imaging. As expected, IR-786 exhibited apparent tumor uptake for 24 h post-injection in the HT-29 xenograft model (Figure 7a). The tumor tissues were finally harvested to reconfirm the fluorescence signals compared to neighboring normal tissue (Figure 7b). Hence, IR-786 showed nonspecific uptake in both the tumor and BAT so that it may not be difficult to distinguish the BAT depending on the tumor location. Most importantly, BF800-AM revealed no tumor accumulation within 24 h after injection in the HT-29 tumor-bearing mice (Figure 7c). Obviously, the tumors on both sides resected from the BF800-AM-treated mice have no fluorescence signals after comparing with adjacent normal tissue (Figure 7d). Moreover, BF800-AM was injected into another tumor type of the NCI-H460 xenograft model to reconfirm the BAT-specific targeting without tumor uptake (Figure 7e). Interestingly, BF800-AM also showed no tumor uptake for 24 h post-injection in the NCI-H460 xenograft model and only high uptake in the BAT. The resected tumor tissue has no fluorescence signal compared with other resected organs (Figure 7f). Therefore, BF800-AM could be successfully used to improve the BAT-specific imaging with reduced nonspecific background uptake through the molecular tuning of IR-786.

## 3. Discussion

Many previous studies suggest that accurate detection and quantification of BAT mass in the body is an important challenge to treat obesity and diabetes for thermogenic energy dissipation. Although noninvasive imaging techniques, including MRI, PET, CT, and optical imaging, to detect BAT with various kinds of imaging agents have been developed, especially, the lack of BAT-specific NIR fluorescence dyes, has limited their applicability. Since no BAT-targeting small molecules, as a known ligand for a BAT-specific biomarker, have yet been reported, it is difficult to design BAT-targeted NIR dyes based on the structure-inherent targeting approach. Alternatively, Zhang et al. previously developed curcumin-based probes for in vivo NIR fluorescence imaging of BAT and browning of WAT through a top-down imaging screening of 38 fluorescent dyes; however, the exact mechanism to identify key functional domains of curcumin analogues is still unclear [5]. In this regard, the rational design method of BAT-targeting NIR dyes for improved physicochemical properties that maintain BAT targetability, but also possess better water solubility and less cytotoxicity, is to utilize the commercially available and chemically modifiable NIR dyes. Thus, the commercially available BAT-targeted NIR dye, IR-786, is selected to use for improved BAT imaging after chemical modification. In the present study, a new type of heptamethine cyanine dye can be readily prepared by incorporating a tyramine linker on the heptamethine bridge of IR-786 to achieve better water solubility and biocompatibility as well as improved BAT imaging.

This study provides a proof of concept that BAT-targeting efficiency of IR-786 can be altered by modulating physicochemical properties, such as hydrophobicity and surface charge. In terms of the surface charge, we demonstrate that the more positively charged BF800-AM shows improved BAT imaging with lower nonspecific background uptake, while the zwitterionic BF800-CA exhibits poor uptake in BAT due to the surface charge compensation for the cationic IR-786. Although the mechanism of targeting BF800-AM to the BAT is yet to be fully understood, the better water solubility and the additional positive charge introduced in the IR-786 structure may play a significant role in BAT-specific uptake and retention, by comparison with that of BF800-CA. Indeed, there are many important factors, such as hydrophobicity, net surface charge, topological polar surface area, and key structural domain, to explain the mechanisms for tissue distribution, accumulation, and elimination characteristics complying with the Lipinski’s rules. However, in vivo behaviors of small molecules are not always consistent with in silico, in vitro, and ex vivo studies. Thus, the better way to identify interesting new phenomena is to set up the target-focused chemical library and collect in vivo data, in spite of the time-consuming and complicated process.

Since IR-786 showed heterogeneous uptake in both BAT and tumor tissue as shown in this study, the BAT-specific or tumor-specific uptake of IR-786 is uncontrollable and indistinguishable when tumor tissues are located at the position adjacent to BAT [10]. Hence, understanding the uptake mechanism of IR-786 in tumor or BAT still remains unclear. Most importantly, it should be highlighted that the structure-modified BF800-AM could selectively target the BAT only, unlike IR-786, without tumor uptake in subcutaneous tumor xenograft models. Although the two similar features of tumor tissue and BAT are the extensive vasculature and high levels of mitochondria, BF800-AM only revealed BAT-selective uptake in such tissue environments. In most cases, the tumor uptake of heptamethine cyanine dyes, such as IR-786, was explained by the involvement of organic anion-transporting polypeptides (OATPs) [13,14]. Recently, Usama et al. demonstrated that a meso-chloride placed on the heptamethine bridge in serum can form a covalent adduct with albumin, which undergoes receptor-mediated endocytosis of albumin, resulting in increased tumor accumulation through the enhanced permeability and retention effect [15,16]. Based on this hypothesis, the BAT-selective uptake of BF800-AM with no tumor uptake in tumor-bearing mice can be explained by the absence of chloro-cyclohexene on the heptamethine skeleton. Therefore, we assume that the appropriate hydrophobicity and molecular surface charge distribution involve translocation of the BF800-AM from the bloodstream to the lipophilic interior of the brown adipocytes.

In the following study, the BAT-targeting efficiency of BF800-AM will be tested with the BAT stimulators such as Norepinephrine and cold exposure, which always induces BAT activation, and also with chronic drug treatment such as the β3-adrenoceptor agonist CL316,243, which is known to activate BAT thermogenesis and used to monitor BAT mass change and browning of WAT [6]. In the case of IR-786, Nakayama et al. previously proved that IR-786 showed a statistically significant increase in BAT perfusion after treatment of such BAT stimulators in both wild-type and transgenic mice [6]. These results suggest that IR-786 targets the mitochondria of brown adipocytes and can be useful for high throughput screening of physiological and pharmacological modulators of BAT and obesity. Therefore, further studies are important to determine the BAT sensitivity of BF800-AM derived from IR-786, by determining whether they are activation-dependent or independent in BAT mass and metabolic state.

In summary, we demonstrated how the molecular tuning of IR-786 can be effective in BAT-targeting to better detect and monitor BAT mass. The newly designed BF800-AM appears to be an ideal NIR dye for BAT that, when used in noninvasive whole-body NIR fluorescence imaging, can lead to an accurate detection and quantification of BAT, regardless of the tumor-bearing or normal mice. This study lays the foundation for future advances that may lead to the discovery of novel small-molecule dyes for improved target-specific imaging with excellent in vivo performance.

## 4. Materials and Methods

### 4.1. Synthesis of BF800-AM and BF800-CA NIR Dyes

Reagents and solvents were purchased from Sigma-Aldrich (St. Louis, MO, USA) and were used without further purification. BF800-AM was synthesized as follows. Before introducing a tyramine linkage on the meso-chlorine atom of commercially available IR-786, tert-butyloxycarbonyl (Boc)-protected tyramine was prepared by adding triethylamine (0.23 g, 2.28 mmol) and Boc anhydride (0.5 g, 2.29 mmol) into tyramine solution (0.21 g, 1.53 mmol) in dimethylformamide (DMF; 5 mL). The reaction mixture was stirred at ambient temperature for 2 h. To the above solution, under nitrogen atmosphere, sodium hydride (0.04 g, 1.6 mmol) was added, and the mixture was stirred at ambient temperature for 1 h. Subsequently, IR-786 (0.06 g, 0.12 mmol) was added to the above solution and the mixture was stirred at ambient temperature for 17 h. For the Boc deprotection, a solution of trifluoroacetic acid (TFA) and water (5 mL, 50/50 *v*/*v*%) was mixed with the above solution and stirred at ambient temperature for an additional 2 h. The solvents were then evaporated from the reaction mixture on rotavapor at 60 °C, and the obtained residue was dried under vacuum. Meanwhile, BF800-CA was synthesized as follows. 3-(4-mercaptophenyl)propionic acid (0.05 g, 0.27 mmol) was added to a solution of IR-786 (0.06 g, 0.12 mmol) in dimethyl sulfoxide (DMSO; 5 mL) under nitrogen atmosphere, and the reaction was stirred at ambient temperature for 1 h. The reaction mixture was crystallized with a mixture of ethyl acetate and diethyl ether, filtered, and dried under vacuum. The crude products were separated using a preparative high-performance liquid chromatography system (Waters, Milford, MA, USA). The molecular weights of the purified products were confirmed by the Dionex UltiMateTM 3000 mass spectrometry system (Thermo Scientific, Waltham, MA, USA).

### 4.2. Optical and Physicochemical Property Analyses

Optical properties of NIR dyes were measured in phosphate-buffered saline (PBS, pH 7.4) or 5% bovine serum albumin (BSA) saline. Absorption spectra of NIR dyes were measured using a fiber optic UV-Vis-NIR (200–1025 nm) spectrometer (Ocean Optics, Dunedin, FL, USA). The molar extinction coefficient (*ε*) was calculated using the Beer–Lambert equation. No absorbance changes in NIR dyes incubated for 72 h at 37 °C were observed in PBS or 5% BSA saline solutions at pH 7.4. Indocyanine green (ICG, Φ = 13% in DMSO) was used as a calibration standard to determine the fluorescence quantum yields of NIR dyes under the conditions of matched absorbance at 770 nm [17,18]. Fluorescence emission spectra of NIR dyes were recorded using a SPARK^®^ 10M microplate reader (Tecan, Männedorf, Switzerland) at excitation wavelengths ranging from 700 to 760 nm and emission wavelengths ranging from 750 to 900 nm. In silico predictions of the distribution coefficient (log*D* at pH 7.4), topological polar surface area (TPSA), surface molecular charge, and hydrogen bond acceptors/donors (HBA/HBD) were performed using Marvin and JChem calculator plugins (ChemAxon, Budapest, Hungary).

### 4.3. In Vitro Cellular Uptake and NIR Fluorescence Microscopy

Mouse embryonic fibroblast NIH/3T3, human colorectal adenocarcinoma HT-29, and human large-cell lung carcinoma NCI-H460 cell lines were obtained from the American Type Culture Collection (ATCC, Manassas, VA, USA). Cells were maintained in Dulbecco’s Modified Eagle Medium (DMEM) or Roswell Park Memorial Institute (RPMI) 1640 media (Gibco BRL, Paisley, UK) supplemented with 10% fetal bovine serum (FBS, Gibco BRL) and an antibiotic-antimycotic solution (Welgene, Daegu, South Korea) in a humidified 5% CO_2_ atmosphere at 37 °C. When the NIH/3T3 cells reached a confluence of approximately 50%, they were rinsed twice with PBS and each NIR dye was added to each well at various concentrations in the range from 2–20 μM. The NIH/3T3 cells were incubated for 24 h at 37 °C and then gently washed with PBS. NIR fluorescence imaging was performed using a Nikon Eclipse Ti-U inverted microscope system (Nikon, Seoul, South Korea). Image acquisition and analysis were performed using the NIS-Elements Basic Research software (Nikon).

### 4.4. In Vitro Cytotoxicity Assay

Cell toxicity and proliferation were evaluated using a 3-(4,5-dimethylthiazol-2-yl)-2,5-diphenyltetrazolium bromide (MTT, Sigma-Aldrich) assay. The NIH/3T3 cells were seeded onto 96-well plates (1 × 10^4^ cells per well). To evaluate the cytotoxicity depending on the dye concentration, the NIH/3T3 cells were treated with each NIR dye (2, 10, 20, and 50 μM) for 1 h and cultured for 24 h after treatment. At each time point, the incubation cell medium was replaced with 100 μL of fresh medium, and 10 μL of the MTT solution was directly added to each 100 μL well. Subsequently, the plates were then incubated for 4 h at 37 °C in a humidified 5% CO_2_ incubator. Finally, the plates were placed in a microplate reader (SPARK^®^ 10M, Tecan) to measure the absorption intensity at 570 nm. Cell viability was calculated using the following formula: cell viability (%) = (*A*_sample_ − *A*_blank_)/(*A*_control_ − *A*_blank_) × 100, where *A* is the average absorbance.

### 4.5. Xenograft Mouse Model

Animal studies were performed in accordance with the guidelines approved by the Chonnam National University Animal Research Committee (CNU IACUC-H-2020-19). Adult (6-week-old, ≈25 g) male athymic nude mice were purchased from OrientBio (Gwangju, South Korea). HT-29 and NCI-H460 cancer cells were respectively cultured and suspended in 100 μL of PBS before being subcutaneously inoculated in the right flank or both sides of each mouse (1 × 10^6^ cells per mouse). When tumor sizes reached approximately 1 cm in diameter, NIR dyes were administered intravenously. Animals were euthanized for in vivo NIR fluorescence imaging within a designated period of time.

### 4.6. In Vivo NIR Fluorescence Imaging

In vivo NIR fluorescence imaging was performed using an FOBI imaging system (NeoScience, Suwon, South Korea). Mice were sacrificed 4 and 24 h after injection of each NIR dye, and their major organs, BAT, and tumors were harvested and imaged to confirm the time-dependent biodistribution of each NIR dye. The fluorescence intensities on the BAT sites and excised organs were analyzed using ImageJ version 1.45q (National Institutes of Health, Bethesda, MD, USA).

### 4.7. Statistical Analysis

Statistical analysis was performed by one-way analysis of variance for multiple comparison tests. The results were represented as mean ± standard deviation (S.D.). A value of *p* < 0.05 was considered statistically significant. Curve fitting was performed using the Prism software (GraphPad, San Diego, CA, USA).

### 4.8. Histological Analysis

Resected BAT was preserved for hematoxylin and eosin (H&E) staining and microscopic observation. The BAT samples were fixed in 4% paraformaldehyde and flash-frozen in an optimal cutting temperature (OCT) compound using liquid nitrogen. Frozen samples were cryosectioned (10 µm thick), observed by fluorescence microscopy, and then stained with H&E. Histological analysis and NIR fluorescence imaging were performed on a Nikon Eclipse Ti-U inverted microscope system (Nikon).

## Data Availability

Not applicable.
